# Myeloma precursor disease (MGUS) among rescue and recovery workers exposed to the World Trade Center disaster

**DOI:** 10.1038/s41408-022-00709-2

**Published:** 2022-08-22

**Authors:** Rachel Zeig-Owens, David G. Goldfarb, Benjamin J. Luft, Xiaohua Yang, Kazunori Murata, Lakshmi Ramanathan, Katie Thoren, Sital Doddi, Urvi A. Shah, Alexandra K. Mueller, Charles B. Hall, Orsi Giricz, Amit Verma, David J. Prezant, Ola Landgren

**Affiliations:** 1 Department of Medicine, Montefiore Medical Center, Bronx, NY USA; 2 Bureau of Health Services, Fire Department of the City of New York, Brooklyn, NY USA; 3 Department of Epidemiology and Population Health, Albert Einstein College of Medicine, Bronx, NY USA; 4 Department of Medicine, Renaissance School of Medicine at Stony Brook University, Stony Brook, NY USA; 5 Department of Pathology and Laboratory Medicine, Memorial Sloan Kettering Cancer Center, New York, NY USA; 6 Myeloma Program, Sylvester Comprehensive Cancer Center, University of Miami, Miami, FL USA; 7 Myeloma Service, Department of Medicine, Memorial Sloan Kettering Cancer Center, New York City, NY USA; 8 The Leukemia & Lymphoma Society, Rye Brook, NY USA; 9 Division of Hemato-Oncology, Department of Oncology, Albert Einstein College of Medicine & Montefiore Medical Center, Bronx, NY USA; 10 Department of Medicine, Division of Pulmonary Medicine, Montefiore Medical Center and Albert Einstein College of Medicine, New York, NY USA; 11 Myeloma Division, Sylvester Comprehensive Cancer Center, University of Miami, Miami, FL USA

**Keywords:** Epidemiology, Risk factors

## Abstract

An elevated risk of myeloma precursor disease, monoclonal gammopathy of undetermined significance (MGUS), was identified among Fire Department of the City of New York (FDNY) World Trade Center (WTC)-exposed firefighters. Further investigation was needed to determine if these findings were reproducible in a more heterogeneous WTC-exposed rescue/recovery workers cohort, the Stony Brook University-General Responder Cohort GRC (SBU-GRC). MGUS risk was compared between the cohorts and to published general population estimates from Olmsted County, MN, USA. In this observational seroprevalence study, odds ratios (OR) and age-standardized risk ratios (RR) of MGUS (M-spike and light-chain-MGUS combined), M-spike, and light-chain-MGUS were estimated using logistic regression. Age-standardized prevalences were calculated for white males aged 50–79; RRs were estimated by comparing risk in the WTC-exposed cohort with the Olmsted County screened cohort. SBU-GRC had elevated odds of MGUS compared with FDNY (OR = 1.38; 95%CI = 1.00–1.89). The age-standardized prevalence of MGUS was 9.0/100 persons (95%CI = 7.5–10.6), over two-fold higher than the general population (RR = 2.08; 95%CI = 1.72–2.51); the age-standardized prevalence of light-chain-MGUS was 3.5-fold higher (RR = 3.54; 95%CI = 2.52–4.97). This study adds to mounting evidence supporting an association between WTC/environmental exposures and MGUS among rescue/recovery workers. Access to MGUS screenings for the entire WTC-exposed cohort could allow for treatment interventions that improve survival.

## Introduction

Multiple myeloma is one of the most common hematologic malignancies among adults with about 35,000 cases diagnosed in 2021 in the United States and an annual incidence rate of 6.7 per 100,000 [1–3]. While mortality has decreased over the years, the current 5-year survival rate is only 58% [4]. Multiple myeloma is a clonal neoplasm of differentiated B cells (plasma cells) typically characterized by abnormal serum immunoglobulins in peripheral blood. It is preceded by a precursor stage known as monoclonal gammopathy of undetermined significance (MGUS), including the subtype, light-chain-MGUS, which can be detected in peripheral blood [5].

Although the cause of multiple myeloma, as well as MGUS and light-chain-MGUS, remain elusive, previous studies have reported an increased risk among individuals exposed to known and suspected carcinogens, including polychlorinated biphenyl (PCB), dioxins, polycyclic aromatic hydrocarbons (PAH), and asbestos [6–8]. The terrorist attacks on the World Trade Center (WTC) on 9/11/2001 (9/11) created an unprecedented environmental exposure to aerosolized dust and gases that contained these compounds and other possible carcinogens [9]. These substances were produced by the collapse and burning of the buildings and by the diesel smoke emitted from heavy equipment used during the 10-month rescue/recovery effort. Cohort studies of WTC-exposed rescue/recovery workers provided possible evidence linking exposure to the WTC aerosolized dust and gases with cancers, including multiple myeloma [10–14]. A small case series (*N* = 8) suggested an excess of early onset multiple myeloma among WTC-exposed first responders in the General Responder Cohort (GRC); 4 cases were 45 years or younger at diagnosis [15]. Previously, we identified and characterized all WTC-exposed white male firefighters from Fire Department of the City of New York (FDNY) diagnosed with multiple myeloma from 9/12/2001 to 7/1/2017; a total of 16 cases were identified with 7 having light-chain multiple myeloma [16]. Since 2011, studies examined the post-9/11 incidence of multiple myeloma, and other cancers, in three WTC-exposed cohorts compared with the general population. These studies found multiple myeloma was elevated in the WTC-exposed rescue/recovery workers compared with the general population. However, in only one study was this association statistically significant [10–13].

We recently screened for MGUS among 781 white male WTC-exposed FDNY firefighters over age 50. We estimated the age-specific prevalence of MGUS/light-chain-MGUS in WTC-exposed FDNY male firefighters and compared the prevalence with published estimates from the Olmsted County, MN comparison population [16]. We also assessed patterns of MGUS in relation to our exposure metric (time of initial arrival at the WTC-site) to test for a possible exposure-response association. We found the age-standardized prevalence rate of MGUS and light-chain-MGUS combined to be 1.8-fold higher than rates from the Olmsted County, MN reference population, and the age-standardized prevalence rate of light-chain-MGUS alone was more than three-fold higher. The results from this initial screening study suggest over time the rate of multiple myeloma may increase in WTC-exposed firefighters as the individuals with MGUS develop multiple myeloma.

The Olmsted County study procedures were the same as described below, and cohorts were racially similar [17], and thus provided a valuable comparison for our study. However, the question of whether the observed association between WTC-exposed firefighters and MGUS was due to WTC exposure or driven by underlying occupational exposures (i.e., firefighting exposure) remained; neither a comparison group comprised exclusively of firefighters with no exposure to the WTC disaster nor a cohort of non-firefighter WTC-exposed individuals were available when we conducted the prior study. Specifically, no other study meeting those criteria screened *all* participants for light-chain-MGUS and MGUS in the same manner that we did. To expand on the previous study, our objectives were to: (1) determine the prevalence of MGUS in the large (*N* = 1197) WTC-exposed Stony Brook University GRC (SBU-GRC) cohort (mostly law enforcement and construction workers) [18] and compare the results to WTC-exposed rescue/recovery workers from FDNY that now includes both firefighters and emergency medical service (EMS) providers; and (2) compare the prevalence in the combined WTC cohort to the non-WTC-exposed, but demographically similar, Olmsted County cohort.

## Materials/subjects and methods

### Study population

The study population is comprised of WTC-exposed rescue/recovery workers from the FDNY and SBU-GRC cohorts. The source population for the FDNY cohort includes firefighters and EMS providers who responded to the WTC disaster, received a medical monitoring exam between 12/2013 through 10/2015, and consented to serum collection for future analyses (*n* = 1498). The source population for the SBU-GRC cohort includes mostly members of law enforcement and construction workers who were exposed to the WTC disaster and consented to have serum collected during medical monitoring exams (*n* = 1197) [18]. Participants with a known diagnosis of multiple myeloma or a related hematologic malignancy (i.e., non-Hodgkin lymphoma, leukemia) prior to their blood draw were excluded (*n* = 32, 16 from each cohort). The final study population included 2663 participants (*n* = 1482 from FDNY and *n* = 1181 from SBU-GRC), after applying this exclusionary criterion. This study was approved by the institutional review boards at Montefiore Medical Center/Albert Einstein College of Medicine and Stony Brook University Medical Center. All participants provided written consent to research.

### Serum specimen and laboratory methods

A 0.5 mL aliquot tube for each study participant was shipped on dry ice to the Protein Immunology Laboratory at Memorial Sloan Kettering Cancer Center where protein assays were performed. Samples were processed between 2013 and 2015 for FDNY participants [16] and 2020 and 2021 for SBU-GRC participants. Additional details regarding collection procedures and lab methods are described elsewhere [19–22]. Briefly, M protein was detected and quantified, and free light chain (FLC) assays were performed. Measurement error of the outcome (i.e., batch effects) was unlikely to result in a bias since all samples were worked up by the same clinical staff and the same protocol within a relatively short period of time. We analyzed serum samples for all participants by using conventional agarose-gel electrophoresis, which revealed the occurrence and pattern (including determination of the size) of M proteins in the study cohort. If there was an abnormal band or equivocal pattern, immunofixation was performed in order to validate and to specify the type of M protein. Then we analyzed FLC levels in all specimens using the Freelite® assay (The Binding Site) on the Optilite® analyzer (The Binding Site).

All results were assessed by two of the authors (O.L. and K.M.) in a blinded fashion. Lab results were categorized as M-spike-MGUS, light-chain-MGUS, and no MGUS. The classical definitions of M-spike-MGUS and light-chain-MGUS used in previous FDNY and Olmsted County prevalence studies were applied [16, 17, 23]. Briefly, light-chain-MGUS was defined as having an abnormal FLC-ratio (FLC-R) (<0.26 or >1.65), the nonexistence of monoclonal protein (i.e., M-spike), elevation of the involved light chain above the appropriate cut-off point, and absence of known myeloma or related hematologic cancer.

### Demographics and other covariates

For FDNY participants, birth date, race, and sex were obtained from employee records; BMI and smoking status were obtained from the monitoring exam at the time of blood draw; self-reported WTC exposure was obtained from the baseline questionnaire. For SBU-GRC participants, birth date, race, sex, and WTC exposure were obtained from baseline questionnaires during monitoring exams; BMI and smoking status were obtained during monitoring exams at the same time as blood collection. Nine participants did not have a weight measurement at the time of blood draw so individual weights were imputed using the mean values from the participants’ monitoring exams directly before and after the draw date. Further details regarding data acquired from each cohort, including the WTC exposure metrics, demographic, and health information are described in greater detail elsewhere [18, 24, 25]. For this study, WTC exposure was classified as a dichotomous variable defined as being caught in the WTC-dust cloud (i.e., arriving at the WTC disaster site on the morning of 9/11/2001) vs not being caught in the WTC dust cloud (i.e., arriving after the morning of 9/11/2001 and thus considered less exposed).

For FDNY, cancer history was ascertained using linkages to Arizona, Connecticut, Florida, New Jersey, New York, North Carolina, Pennsylvania, South Carolina, and Virginia state cancer registries (99% of FDNY cohort resided in these states) as well as via questionnaire data and self-reports, which were confirmed using medical records and were reviewed by a trained clinician [26]. For the SBU-GRC cohort, history of cancer data were obtained via linkages with New York, New Jersey, Pennsylvania, Connecticut, and Florida (99% of SBU-GRC cohort resided in these states) [14].

### Comparison population: Olmsted County, Minnesota

The comparison population for the external analyses used published data from the population-based Olmsted County, Minnesota study [17]. To date, this is the only other known study to screen for both M-spike-MGUS and light-chain-MGUS using the same methods detailed above. The racial make-up of the Olmsted County population is largely white, similar to the WTC-exposed cohorts. As done in our first study, to improve comparability with the analytic population, the Olmsted County cohort was again restricted to males, 50–79 years old (*N* = 7612).

### Statistical analysis

For participants with more than one sample drawn (*n* = 73 for FDNY; *n* = 20 for SBU-GRC) during the study period, the most recently drawn sample was used for analyses. Demographic and other characteristics were initially assessed as counts and proportions. Statistics of central tendency and normality were assessed graphically and using the Shapiro–Wilk test.

Logistic regression was used to compare WTC-exposed FDNY and SBU-GRC responders. Models included sex, age at blood draw (using a multiplier of 10-years), BMI (normal as 18.5–24.9 kg/m^2^; overweight as 25.0–29.9 kg/m^2^; obese as ≥30 kg/m^2^), and self-reported smoking status (never, current or former) as covariates. These potential confounders were selected a priori based on a review of the literature [27, 28]. Three outcomes were evaluated separately: overall MGUS; and each subtype: M-spike-MGUS; light-chain-MGUS. The cohorts were subsequently pooled to evaluate the effect of WTC dust cloud exposure on each outcome. Eight participants for whom we had no exposure information were excluded from this analysis only.

Crude age-specific prevalence rates were calculated for white males only as the total number of cases within each age stratum divided by the total number of individuals within that age stratum. Prevalence rates for overall MGUS, M-spike-MGUS, and light-chain-MGUS were calculated for the combined study population, as well as for each cohort separately. Participants older than 79 years were excluded from this analysis due to small numbers in the FDNY cohort. Additionally, to permit external comparison, prevalence rates were age-standardized to the US 2000 male population for ages 50–79 years using 10-year age bands, and 95% confidence intervals were calculated for age-standardized risks using the modified γ approximation method, which assumes a Poisson distribution. The WTC-exposed cohorts were then compared to Olmsted County with standardized risk ratios (RRs). Standard errors for 95% Mantel-Haenszel confidence limits of RRs were calculated using the Greenland and Robins variance formula [29]. All analyses were performed using SAS, version 9.4.

## Results

### Demographic and other characteristics for FDNY and SBU-GRC

The analytic cohort included 2663 participants overall (1482 from FDNY and 1181 from SBU-GRC). Table 1 shows selected demographic characteristics of participants from FDNY and SBU-GRC. The crude prevalence for overall MGUS was 5.9 and 7.9% for FDNY and SBU-GRC participants, respectively, and 6.8% for the SBU-GRC and FDNY combined cohort, 82.9% of the cases were kappa predominant. Both FDNY and SBU-GRC participants were predominantly white males aged 50–59. The mean age at time of specimen collection was 55.2 years (standard deviation [SD] = 8.7) for the overall cohort and 54.4 (SD = 9.4) and 56.2 (SD = 7.7) for the FDNY and SBU-GRC participants, respectively. The mean age of participants with either MGUS subtype was 60.2 (SD = 8.5) and 60.0 (SD = 8.4) and 60.4 (SD = 8.6) for M-spike-MGUS and light-chain-MGUS, individually. The cohorts had similar proportions of responders that were exposed to the dust cloud (17.9% for FDNY and 19.2% for SBU-GRC). Nearly half of the cohort was obese (49.9%), with GRC-SBU having a slightly higher proportion with a BMI > 30 (53.8% vs. 46.8%). Clinical characteristics of the MGUS cases are found in the Supplements (S1 and S2).

**Table 1:** Selected characteristics by cohort.

	FDNY (*n* = 1482)	SBU-GRC (*n* = 1181)	All participants (*n* = 2663)
Sex			
Male	1423 (96.0)	1134 (96.0)	2557 (96.0)
Female	59 (4.0)	47 (4.0)	106 (4.0)
Age at blood draw			
30–39	88 (5.9)	4 (0.3)	92 (3.5)
40–49	397 (26.8)	237 (20.1)	634 (23.8)
50–59	612 (41.3)	603 (51.1)	1215 (45.6)
60–69	293 (19.8)	271 (22.9)	564 (21.2)
70–79	88 (5.9)	63 (5.3)	151 (5.7)
80+	4 (0.3)	3 (0.3)	7 (0.3)
Race/ethnicity			
White	1226 (82.7)	1087 (92.0)	2313 (86.9)
Black	114 (7.7)	16 (1.4)	130 (4.9)
Hispanic	122 (8.2)	63 (5.3)	185 (6.9)
Asian	15 (1.0)	7 (0.6)	22 (0.8)
Other	5 (0.3)	8 (0.7)	13 (0.5)
BMI category			
Normal (18.5–24.9)	161 (10.9)	78 (6.6)	239 (9.0)
Overweight (25.0–29.9)	621 (41.9)	468 (39.6)	1089 (40.9)
Obese (≥30)	600 (47.2)	635 (53.8)	1335 (50.1)
Smoking			
Current	71 (4.8)	51 (4.3)	122 (4.6)
Former	519 (35.0)	412 (34.9)	931 (35.0)
Never	891 (60.1)	718 (60.8)	1609 (60.4)
Missing	1 (0.1)	0 (0.0)	1 (0.0)
Dust cloud exposure			
Yes	266 (17.9)	227 (19.2)	493 (18.5)
No	1216 (82.1)	946 (80.1)	2162 (81.2)
Missing	0 (0.0)	8 (0.7)	8 (0.3)
MGUS			
Overall MGUS	88 (5.9)	93 (7.9)	181 (6.8)
M-spike-MGUS	51 (3.4)	52 (4.4)	103 (3.9)
Light-chain MGUS	37 (2.5)	41 (3.5)	78 (2.9)

Some percentages may not add to 100 due to rounding. *BMI* Body Mass Index, *FDNY* Fire Department of the City of New York, *SBU-GRC* Stony Brook University General Responder Cohort, *MGUS* Monoclonal Gammopathy of Undetermined Significance.

### Logistic regression models comparing WTC exposure (FDNY vs SBU-GRC; Dust cloud vs. later arrivals)

Counts/proportions of overall MGUS, M-spike-MGUS, and light-chain-MGUS, as well as logistic regression models, are displayed in Table 2. After controlling for confounders, a 38% higher odds of overall MGUS was observed for SBU-GRC participants when compared with FDNY participants (OR = 1.38; 95% CI = 1.00–1.89). A similar trend was observed for M-spike-MGUS and light-chain-MGUS. Age, black race, current smoking, and obesity were all positively associated with having overall MGUS and each subtype. Logistic models evaluating dust cloud exposure vs arriving later (considered less exposed) and MGUS did not show significantly elevated odds of MGUS after controlling for confounders (Table 3).

**Table 2:** Logistic regression comparing Stony Brook University General Responder with Fire Department of the City of New York World Trade Center-exposed cohorts.

	Analytic cohort	Overall MGUS	OR (95% CI)	M-spike- MGUS	OR (95% CI)	Light-chain-MGUS	OR (95% CI)
	2663 (100.0)	181 (100.0)		103 (100.0)		78 (100.0)	
*Cohort n (%)*							
SBU-GRC	1181 (44.3)	93 (51.4)	1.38 (1.00, 1.89)	52 (50.5)	1.27 (0.85, 1.92)	41 (52.6)	1.48 (0.92, 2.38)
FDNY	1482 (55.7)	88 (48.6)	Ref	51 (49.5)	Ref	37 (47.4)	Ref
*Sex n (%)*							
Male	2557 (96.0)	173 (95.6)	1.05 (0.49, 2.26)	100 (97.1)	1.49 (0.45, 4.89)	73 (93.6)	0.78 (0.30, 2.08)
Female	106 (4.0)	8 (4.4)	Ref	3 (2.9)	Ref	5 (6.4)	Ref
Age at blood draw mean (SD)*	55.2 (8.7)	60.2 (8.5)	2.00 (1.67, 2.40)	60.0 (8.4)	1.85 (1.47, 2.34)	60.4 (8.6)	2.05 (1.57, 2.67)
*Race n (%)*							
White	2313 (86.9)	148 (81.8)	Ref	88 (85.4)	Ref	60 (76.9)	Ref
Black	130 (4.9)	17 (9.4)	2.64 (1.48, 4.68)	8 (7.8)	2.00 (0.91, 4.38)	9 (11.5)	3.23 (1.48, 7.02)
Hispanic	185 (6.9)	13 (7.2)	1.35 (0.74, 2.48)	6 (5.8)	1.03 (0.44, 2.41)	7 (9.0)	1.77 (0.78, 4.01)
Asian	22 (0.8)	1 (0.6)	1.24 (0.16, 9.43)	1 (1.0)	2.10 (0.27, 16.09)	0 (0.0)	n/a
Other	13 (0.5)	2 (1.1)	1.70 (0.34, 8.44)	0 (0.0)	n/a	2 (2.6)	4.61 (0.93, 22.91)
*BMI category n (%)*							
Normal (18.5–24.9)	239 (9.0)	18 (9.9)	Ref	10 (9.7)	Ref	8 (10.3)	Ref
Overweight (25.0–29.9)	1089 (40.9)	63 (34.8)	0.81 (0.46, 1.42)	39 (37.9)	0.91 (0.44, 1.87)	24 (30.8)	0.72 (0.31, 1.64)
Obese (≥30)	1335 (50.1)	100 (55.2)	1.15 (0.67, 1.97)	54 (52.4)	1.09 (0.54, 2.21)	46 (59.0)	1.21 (0.56, 2.66)
*Smoking n (%)*							
Current	122 (4.6)	10 (5.5)	1.48 (0.74, 2.96)	5 (4.9)	1.36 (0.53, 3.50)	5 (6.4)	1.56 (0.60, 4.08)
Former	931 (35.0)	78 (43.1)	1.14 (0.82, 1.58)	48 (46.6)	1.35 (0.89, 2.06)	30 (38.5)	0.89 (0.54, 1.47)
Never	1609 (60.4)	93 (51.4)	Ref	50 (48.5)	Ref	43 (55.1)	Ref
Missing	1 (0.0)	0 (0.0)	n/a	0 (0.0)	n/a	0 (0.0)	n/a

Models control for sex, age at blood draw, race, BMI, and smoking status; *OR for age calculated using 10-year increase; Some percentages may not add to 100 due to rounding. *BMI* Body Mass Index, *FDNY* Fire Department of the City of New York, *SBU-GRC* Stony Brook University General Responder Cohort, *MGUS* Monoclonal Gammopathy of Undetermined Significance.

**Table 3:** Logistic regression evaluating World Trade Center dust cloud exposure.

	Overall MGUS		M-spike-MGUS		Light-chain-MGUS	
Dust cloud exposure	*n* (%)	OR (95% CI)	*n* (%)	OR (95% CI)	*n* (%)	OR (95% CI)
Yes	36 (7.3)	1.15 (0.78, 1.70)	22 (4.5)	1.28 (0.79, 2.10)	14 (2.8)	0.99 (0.54, 1.80)
No	143 (6.6)	Ref	80 (3.7)	Ref	63 (2.9)	Ref

Models include sex, age at blood draw, race, BMI, and smoking status as covariates. 8 participants with missing exposure data were excluded from this analysis.

*MGUS* Monoclonal Gammopathy of Undetermined Significance.

### Age-standardized prevalence rates and risk ratios compared with the Olmsted County comparison population

**Fig. 1:**
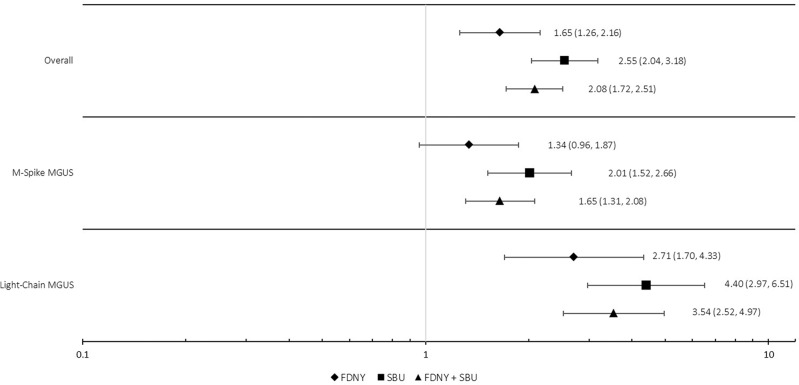
Age standardized risk ratios among white, male participants, aged 50–79 compared with the comparison population from Olmsted County, MN, USA [17]. Reference is demographically similar Olmsted county male participants aged 50–79; all rates were first age-standardized to the US 2000 population; diamonds represent FDNY participants, squares: SBU participants; triangles represent all World Trade Center exposed participants (FDNY and SBU-GRC); FDNY Fire Department of the City of New York, SBU-GRC Stony Brook University General Responder Cohort, MGUS Monoclonal Gammopathy of Undetermined Significance.

Our external analysis comparing prevalence to the Olmsted County comparison population included white male participants aged 50–79, only. Prevalence rates standardized to the US 2000 population were 9.0, 5.5, and 3.5% for the SBU-GRC and FDNY combined cohort for overall MGUS, M-spike-MGUS, and light-chain-MGUS, respectively. Supplemental Table S3 displays all crude risks as well as age-standardized prevalences to the US 2000 population. Figure 1 demonstrates age-standardized RRs for overall MGUS (a), M-spike-MGUS (b), and light-chain-MGUS (c). A two-fold higher risk of overall MGUS (RR = 2.08; 95% CI = 1.72–2.51) was observed for the combined FDNY and SBU-GRC compared to the Olmsted County comparison population. This result was higher for light-chain-MGUS (RR = 3.54; 95% CI = 2.52–4.97). SBU-GRC participants had a slightly higher risk than FDNY for each outcome and significantly higher risk than Olmsted County participants.

## Discussion

In this large comprehensive study focusing on prevalence of MGUS, the precursor for myeloma, among WTC-exposed rescue/recovery workers from the SBU-GRC and FDNY cohorts, we observed striking patterns. Among WTC-exposed male rescue/recovery workers aged 50–79, we observed an over two-fold elevated age-standardized risk of overall MGUS (RR = 2.08; 95% CI = 1.72–2.51) when compared with demographically similar participants from Olmsted County, MN. As we saw in our earlier study [16], the risk was greatest for the light-chain-MGUS subtype: over 3.5-fold greater risk for light-chain-MGUS (RR = 3.54; 95% CI = 2.52–4.97) and 1.65 times greater for M-spike-MGUS (RR = 1.65; 95% CI = 1.31–2.08). Further, our internal analyses demonstrated a significantly higher risk for overall MGUS for the SBU-GRC cohort when compared with the FDNY cohort, after controlling for sex, age at blood draw, race, BMI, and smoking. Together, these findings add to a growing body of evidence that support a relationship between exposure to the WTC disaster site and myeloma precursor disease.

In our initial study, which evaluated the association between WTC exposure and MGUS among white male firefighters only, we observed a 1.8-fold significantly higher age-standardized risk of overall MGUS and an over three-fold higher risk of light-chain-MGUS when compared with the Olmsted County, MN comparison cohort [16]. Further, in the myeloma case series analysis of the WTC-exposed firefighter study, age of disease onset occurred, on average, twelve years earlier than what is observed in the general population (57 vs 69 years), with 71% of participants having CD20-expressing plasma cells—characteristics associated with a poorer prognosis. Here, we were motivated to confirm our investigations among WTC-exposed individuals who were non-firefighters by expanding our work to study FDNY EMS providers and general responders, such as law enforcement and construction workers from the SBU-GRC, as well.

Many WTC-exposed rescue/recovery workers were initially exposed to aerosolized dust and toxic fumes from burning jet fuel and building materials. WTC-exposed members of the SBU-GRC had comparable levels of self-reported exposure to the toxic dust cloud as the FDNY cohort (19% vs 18%), and many workers endured continued exposure throughout the clean-up effort which ended the summer of 2002. Despite the similar level of self-reported WTC exposure, we found the SBU-GRC participants had a greater risk of MGUS than the FDNY participants. The exact underlying causes for the observed higher prevalence among SBU-GRC participants remain largely unclear and may be confounded. For example, it is plausible that this sub-sample of SBU-GRC participants had higher levels of sustained exposure than FDNY rescue/recovery workers that were not captured by our self-reported exposure metric. Although the distribution of known risk factors between SBU-GRC and FDNY rescue/recovery workers were comparable (Table 1), potential explanations for the increased risk include unmeasured confounding of environmental exposures, prior occupational exposures, and baseline health behaviors that are not accounted for by smoking and BMI. Another possibility is that the SBU-GRC samples were drawn approximately five years after the FDNY samples. This additional time since 9/11 may have resulted in a more pronounced WTC signal. The observed elevated risk between the two cohorts may also be a reflection of an extended latency period from exposure to disease onset. Further investigations are needed to better understand the observed higher prevalence among SBU-GRC participants. Nonetheless, given the overall burden of disease in this cohort and the estimated conversion rate of 1% per year from MGUS to myeloma [30], it will be important to monitor both cohorts carefully.

This study has numerous strengths. First, it is the largest known MGUS prevalence study of both FDNY and non-FDNY WTC-exposed responders, and findings were highly reproducible across two distinctive cohorts with varied occupations. Second, methodologies including specimen collection, analytic, and laboratory techniques were highly standardized and consistent for the inaugural FDNY firefighter study, the present study, and the Olmsted County, MN study, to which both WTC studies were compared.

This study was not without limitations. First, we could not establish incidence due to the cross-sectional nature of the data collection. Additionally, samples were drawn between 12 and 18 years after 9/11, and thus the latency period between exposure and onset of the disease is unclear. Follow-up studies analyzing specimens collected shortly after 9/11 and longitudinally will be essential for understanding both the incidence and latency of myeloma precursor disease, as well as the clinical course of patients who advance to myeloma. By drawing samples from both FDNY and SBU-GRC at the same time, the observed difference between the two groups could be understood. Second, we could not measure important confounders, in particular, competing occupational exposures unrelated to the WTC disaster, as well as other environmental exposures in New York, NY and Long Island, NY. We note that the healthy worker effect may have biased results toward the null. Third, this study was underpowered to detect an exposure-response gradient association between WTC exposure and MGUS. Dust cloud exposure showed a suggestion of increased risk for M-spike-MGUS; however, this result was not statistically significant. Fourth, while the Olmsted County cohort was the best available comparison population that screened for MGUS, participants likely have a different exposure profile given that it is considerably more rural than the greater New York region. Fifth, the concept of free light-change assay drift cannot be ruled out for some of the effect size. Among MGUS samples in the Olmsted County and WTC studies, 64.9 and 82.9% were kappa predominant, respectively. Unfortunately, we don’t have repeated measurements over time for the WTC cohort and thus were unable to adequately address the concept of FLC assay drift, but this warrants future investigation. Our inability to address this is a limitation of the work, however, we highlight that this method is standard in clinical practice as well as in the majority of other studies on this topic. Finally, while this finding is reproducible with regard to occupation, generalizability of these findings to other demographic subgroups such as non-White races and females is lacking. Enhanced screening for MGUS in an expanded cohort would be important for determining other susceptible groups.

In summary, we report a doubling in risk of overall MGUS and an over 3.5-fold elevated risk of light-chain-MGUS suggesting unambiguous associations between environmental exposures present at the WTC disaster site and myeloma precursor disease. Recently, it was reported that rescue/recovery worker cancer patients enrolled in a WTC Health Program had improved survival relative to the New York state population, potentially due to reduced barriers to systematic health surveillance and treatment and no out-of-pocket medical care costs [31]. While a randomized controlled trial examining the risks and benefits of MGUS screening is ongoing and will provide clearer guidance on public health recommendations [32], if improved survival among MGUS screened cohorts is demonstrated, the important findings from our current study provide evidence that screening of WTC-exposed cohorts should be recommended. Further, through screening, we will both better understand the burden of MGUS and further augment survival benefits for this cohort.

## Supplementary information

Supplemental tables

## Data Availability

The datasets generated during and/or analyzed during the current study are available from the corresponding author on reasonable request.
