# Supplementary material for: Myeloma precursor disease (MGUS) among rescue and recovery workers exposed to the World Trade Center disaster
Source: Blood Cancer J. 2022 Aug 22;12(8):120. doi: 10.1038/s41408-022-00709-2 (PMC9395354; doi:10.1038/s41408-022-00709-2)
Supplement: Supplementary file 1 — Supplemental tables [file 41408_2022_709_MOESM1_ESM.docx]

Supplemental Tables:

Table S1: Clinical characteristics of MGUS

|  | **FDNY** | | **SBU-GRC** | | **Total** | |
| --- | --- | --- | --- | --- | --- | --- |
|  | n | % | n | % | n | % |
| Overall MGUS | 88 | 5.9 | 93 | 7.9 | 181 | 6.8 |
| M-spike MGUS | 51 | 3.4 | 52 | 4.4 | 103 | 3.9 |
| IgG | 34 | 2.3 | 32 | 2.7 | 66 | 2.5 |
| IgA | 9 | 0.6 | 7 | 0.6 | 16 | 0.6 |
| IgM | 5 | 0.3 | 10 | 0.8 | 15 | 0.6 |
| Biclonal^a^ | 2 | 0.1 | 2 | 0.2 | 4 | 0.2 |
| Oligoclonal^b^ | 1 | 0.1 | 1 | 0.1 | 2 | 0.1 |
| Light-chain MGUS | 37 | 2.5 | 41 | 3.5 | 78 | 3.0 |

1. N=1 participant with IgG K/IgG L, n=1 with double IgG L, n=1 with IgA K and IgM K, n=1 with double IgG K
2. Multiple small bands

Abbreviations: FDNY, Fire Department of the City of New York; SBU-GRC, Stony Brook University General Responder Cohort, MGUS, Monoclonal Gammopathy of Undetermined Significance

Table S2: Concentration of MGUS

|  | **FDNY** | | **SBU-GRC** | | **Total** | |
| --- | --- | --- | --- | --- | --- | --- |
| Concentration | Median (Q1, Q3) | | Median (Q1, Q3) | | Median (Q1, Q3) | |
| MGUS^a^ |  |  |  |  |  |  |
| M-protein g/dL | 0.33 | 0.20, 0.53 | 0.30 | 0.21, 0.43 | 0.31 | 0.20, 0.48 |
| Light-chain MGUS^b^ |  |  |  |  |  |  |
| Free-κ light chain, mg/dL | 2.44 | 2.16, 3.29 | 2.83 | 2.37, 4.01 | 2.61 | 2.24, 3.55 |
| Free-λ light chain, mg/dL | 1.24 | 1.07, 1.63 | 1.45 | 1.21, 1.79 | 1.36 | 1.14, 1.72 |
| dFLC, mg/dL | 1.26 | 0.98, 1.77 | 1.35 | 1.12, 2.04 | 1.30 | 1.02, 1.92 |
| FLC-ratio, median | 1.92 | 1.77, 2.26 | 2.02 | 1.79, 2.28 | 1.98 | 1.78, 2.26 |

1. Based on those whose M-protein was quantifiable (N=103)
2. Based on those who had LC-MGUS (N=78)

Abbreviations: FDNY, Fire Department of the City of New York; SBU-GRC, Stony Brook University General Responder Cohort, MGUS, Monoclonal Gammopathy of Undetermined Significance;

| **Overall MGUS** | | | | | | | | | | | | |
| --- | --- | --- | --- | --- | --- | --- | --- | --- | --- | --- | --- | --- |
|  | FDNY | | | SBU-GRC | | | FDNY and SBU-GRC combined | | | Olmsted County, MN | | |
| Age group | n | total | % | n | total | % | n | total | % | n | total | % |
| 50-59 | 22 | 523 | 4.2 | 44 | 531 | 8.3 | 66 | 1,054 | 6.3 | 95 | 3,450 | 2.8 |
| 60-69 | 18 | 240 | 7.5 | 21 | 247 | 8.5 | 39 | 487 | 8.0 | 122 | 2,554 | 4.8 |
| 70-79 | 10 | 76 | 13.2 | 12 | 58 | 20.7 | 22 | 134 | 16.4 | 116 | 1,608 | 7.2 |
| Age standardized risk (95% CI) | 50 | 839 | 7.2 (5.2, 9.2) | 77 | 836 | 11.1 (8.6, 13.6) | 127 | 1,675 | 9.0 (7.5, 10.6) | 333 | 7,612 | 4.3 (3.9, 4.8) |
|  |  | | |  | | |  | | |  |  |  |
| **M-spike-MGUS** | | | | | | | | | | | | |
|  | FDNY | | | SBU-GRC | | | FDNY and SBU-GRC combined | | | Olmsted County, MN | | |
| Age group | n | total | % | n | total | % | n | total | % | n | total | % |
| 50-59 | 11 | 523 | 2.1 | 25 | 531 | 4.7 | 36 | 1,054 | 3.4 | 73 | 3,450 | 2.1 |
| 60-69 | 14 | 240 | 5.8 | 12 | 247 | 4.9 | 26 | 487 | 5.3 | 97 | 2,554 | 3.8 |
| 70-79 | 6 | 76 | 7.9 | 8 | 58 | 13.8 | 14 | 134 | 10.5 | 88 | 1,608 | 5.5 |
| Age standardized risk (95% CI) | 31 | 839 | 4.5 (2.8, 6.2) | 45 | 836 | 6.8 (4.3, 9.2) | 76 | 1675 | 5.5 (3.5, 7.6) | 258 | 7,612 | 3.4 (3.0, 3.8) |
|  |  | | |  | | |  | | |  |  |  |
| **Light-chain MGUS** | | | | | | | | | | | | |
|  | FDNY | | | SBU-GRC | | | FDNY and SBU-GRC combined | | | Olmsted County, MN | | |
| Age group | n | total | % | n | total | % | n | total | % | n | total | % |
| 50-59 | 11 | 523 | 2.1 | 19 | 531 | 3.6 | 30 | 1,054 | 2.9 | 22 | 3,450 | 0.6 |
| 60-69 | 4 | 240 | 1.7 | 9 | 247 | 3.6 | 13 | 487 | 2.7 | 25 | 2,554 | 1.0 |
| 70-79 | 4 | 76 | 5.3 | 4 | 58 | 6.9 | 8 | 134 | 6.0 | 28 | 1,608 | 1.7 |
| Age standardized risk (95% CI) | 19 | 839 | 2.7 (1.5, 3.9) | 32 | 836 | 4.3 (2.8, 5.8) | 51 | 1,675 | 3.5 (2.5, 4.4) | 75 | 7,612 | 1.0 (0.8, 1.2) |

Table S3: Prevalence of MGUS among white, male participants aged 50-79

Abbreviations: FDNY, Fire Department of the City of New York; SBU-GRC, Stony Brook University General Responder Cohort, MGUS, Monoclonal Gammopathy of Undetermined Significance

Table S1: Clinical characteristics of MGUS

1. N=1 participant with IgG K/ IgG L, n=1 with double IgG L, n=1 with IgA K and IgM K, n=1 with double IgG K
2. Multiple small bands

abbreviations: FDNY, Fire Department of the city of New York; SBU-GRC, Stony Brook University General Responder Cohort, MGUS, Monoclonal Gammopathy of Undetermined Significance

Table S2: Concentration of MGUS

1. Based on those whose M-protein was quantifiable (N=103)
2. Based on those who had LC-MGUS (N=78)

Abbreviations: FDNY, Fire Department of the city of New York; SBU-GRC, Stony Brook University General Responder Cohort, MGUS, Monoclonal Gammopathy of Undetermined Significance;

Table S3: Prevalence of MGUS among white, male participants aged 50-79

Abbreviations: FDNY, Fire Department of the city of New York; SBU-GRC, Stony Brook University General Responder Cohort, MGUS, Monoclonal Gammopathy of Undetermined Significance
